# Abdominal distension in ICU patient

**DOI:** 10.11604/pamj.2019.33.204.13334

**Published:** 2019-07-15

**Authors:** Ali Jad Abdelwahab Yousef

**Affiliations:** 1 Surgery Department, Faculty of Medicine, Mutah University, Karak, Jordan

**Keywords:** Colonic pseudo obstruction, ogilvie´s syndrome, abdominal distension

## Image in medicine

A 77 year old male, who is known to be diabetic and hypertensive, underwent left hip replacement three months ago. He developed major complications including infections and pulmonary embolism for which he is still hospitalized. Three days ago he became tachypnic, tachycardic, hyopotensive and collapsed suddenly after development of massive abdominal distension with opstipation. He was intubated and surgical consultation sought. His physical examination disclosed a huge distended tense abdomen with tympanic note, absent bowel sounds and empty dilated rectum. He was afebrile with mild leukocytosis .Presence of peritonitis could not be assessed because the patient was unconscious. Portable plain abdomen X-ray was obtained (A). The patient diagnosis was acute colonic pseudo-obstruction (ACPO), also known as Ogilvie's syndrome, which has the clinical and radiological pictures of colonic obstruction without mechanical cause. The patient was treated surgically for fear of imminent perforation. The whole colon was massively dilated but there was no perforation. Cecostomy was done and the bowel was decompressed and the patient improved significantly. He died after one month due to co morbidities. In mechanical obstruction such as volvulus, the course will be more dramatic with rapid progression towards perforation and peritonitis. In ischemic colitis the patient prominent complaint will be severe pain associated with bloody diarrhea. On the other hand, in toxic megacolon the patient will be one, who is acutely ill, febrile, with bloody diarrhea and leukocytosis.

**Figure 1:**
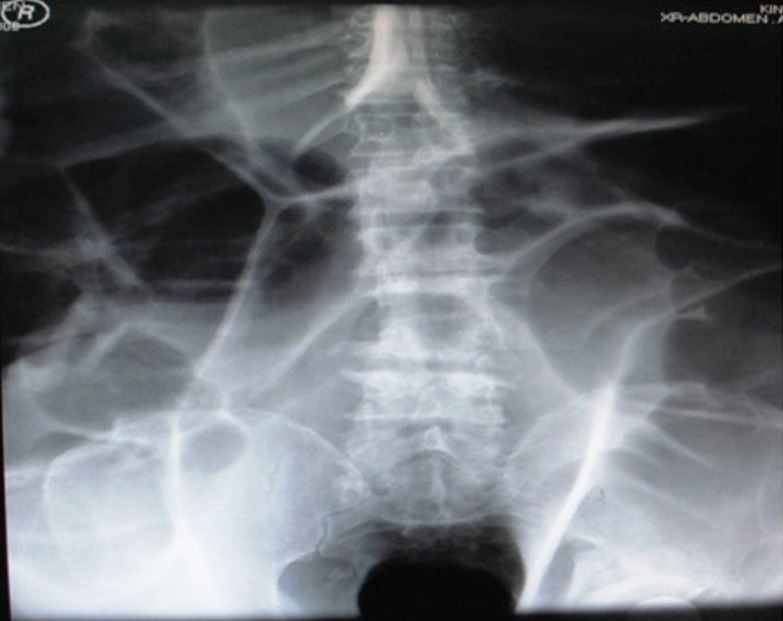
Portable plain abdomen X-ray showing grossly hugely dilated colon with bowel wall edema. The lateral walls are not seen due to huge distension

